# The importance of early arthroscopy in athletes with painful cartilage lesions of the ankle: a prospective study of 61 consecutive cases

**DOI:** 10.1186/1749-799X-1-4

**Published:** 2006-09-28

**Authors:** Christer G Rolf, Caroline Barclay, Masoud Riyami, John George

**Affiliations:** 1 The Sheffield Centre of Sports Medicine, School of Biomedical Sciences, University of Sheffield, 5 Broomfield Road, S10 2SE, Sheffield, UK; 2 Department of Biomedical Imaging, University of Malaya, Malaysia

## Abstract

**Background:**

Ankle sprains are common in sports and can sometimes result in a persistent pain condition.

**Purpose:**

Primarily to evaluate clinical symptoms, signs, diagnostics and outcomes of surgery for symptomatic chondral injuries of the talo crural joint in athletes. Secondly, in applicable cases, to evaluate the accuracy of MRI in detecting these injuries. Type of study: Prospective consecutive series.

**Methods:**

Over around 4 years we studied 61 consecutive athletes with symptomatic chondral lesions to the talocrural joint causing persistent exertion ankle pain.

**Results:**

43% were professional full time athletes and 67% were semi-professional, elite or amateur athletes, main sports being soccer (49%) and rugby (14%). The main subjective complaint was exertion ankle pain (93%). Effusion (75%) and joint line tenderness on palpation (92%) were the most common clinical findings. The duration from injury to arthroscopy for 58/61 cases was 7 months (5.7–7.9). 3/61 cases were referred within 3 weeks from injury. There were in total 75 cartilage lesions. Of these, 52 were located on the Talus dome, 17 on the medial malleolus and 6 on the Tibia plafond. Of the Talus dome injuries 18 were anteromedial, 14 anterolateral, 9 posteromedial, 3 posterolateral and 8 affecting mid talus. 50% were grade 4 lesions, 13.3% grade 3, 16.7% grade 2 and 20% grade 1. MRI had been performed pre operatively in 26/61 (39%) and 59% of these had been interpreted as normal. Detection rate of cartilage lesions was only 19%, but subchondral oedema was present in 55%. At clinical follow up average 24 months after surgery (10–48 months), 73% were playing at pre-injury level. The average return to that level of sports after surgery was 16 weeks (3–32 weeks). However 43% still suffered minor symptoms.

**Conclusion:**

Arthroscopy should be considered early when an athlete presents with exertion ankle pain, effusion and joint line tenderness on palpation after a previous sprain. Conventional MRI is not reliable for detecting isolated cartilage lesions, but the presence of subchondral oedema should raise such suspicion.

## Background

Ankle injuries make up 15% of all sports injuries, sprains constituting 75% out of these cases [1]. While there are accurate clinical tests to evaluate complete ligament ruptures after sprains, associated cartilage injuries are challenging to diagnose, due to the vague nature of their symptoms as stated in the literature, [2] and are therefore often missed [3]. In contact sports such as professional soccer and rugby, the "sprain" mechanism so often referred to can be challenged since it is often blurred in a tackle, which may well involve direct impact from the opponent's leg, shoe or studs. This has not been systematically studied, but was seen in some of the present cases, occurring during games which were filmed by Sky Sports, videos brought by the Team medics. Traditionally, chondral injuries of the ankle are associated with inversion or eversion sprains and ligament tears. In 1991, Taga et al operated 31 patients suffering from lateral ligament injuries and unstable ankles. He revealed that 89% of the acutely injured ankles and 95% of chronic injured ankles also had chondral injuries [4]. These results are consistent with those of Hintermann [1], Schafer [5] and Takao [6], who found high incidences of cartilage lesions in patients with chronic unstable ankles. In none of the present cases, instability was a problem. If a comprehensive initial diagnosis is not made, an athlete can be left with a painful ankle or inability to participate in sport despite thorough rehabilitation. There is no systematic data categorizing characteristic symptoms and signs which refers to cartilage injuries in such cases. It is obvious that the physician may miss these injuries if the suspected mechanism is a simple "sprain" and the symptoms and signs are non-specific. It is well recognized that arthroscopy provides an excellent evaluation and treatment tool for many of these conditions, but usually it is instituted after a substantial delay when symptoms persists despite long term conservative treatments [3,7,8]. MRI of the articular cartilage of the ankle joint is challenging. The cartilage of the distal Tibia plafond and Talus dome measure only 0.4–2.1 mm in thickness, making it challenging to detect surface abnormalities [5,10]. Artefacts are commonly encountered, adding another dimension of complexity [9]. There is a number of MRI sequences available that are promoted as cartilage specific. However, in daily MRI practice, at least in the UK, cartilage specific pulse sequences are rarely used. Instead, a combination of spin-echo, fat-suppressed fast spin-echo, and short inversion time inversion recovery (STIR) images are relied upon. There are a number of studies involving MRI and cartilage injuries in the knee joint, although the majority of studies involve fewer than 50 injuries. Recently, Rubin et al demonstrated that the presence of subchondral oedema in the knee may be indicative of a defect in the overlying articular surface [11,12]. Rubin encouraged that when these focal marrow abnormalities are seen, the overlying cartilage should be inspected carefully with arthroscopy. To our knowledge there is no corresponding study on the ankle.

The purpose of this prospective study was primarily to identify and categorize characteristic clinical symptoms and signs associated with chondral injuries, and secondly to evaluate outcomes of surgery in athletes with persistent exertion ankle pain caused by chondral lesions to the talocrural joint. We did not initially include MRI in our investigation, but since a number of patients brought with them "normal" MRIs, organised by their clubs, we chose to collect and retrospectively re-evaluate the accuracy of these.

## Methods

Our Sports Injury Centre receives referrals from clubs all over the UK. Most professional clubs have their own Team medics who deal with the acute management and initial investigations. Usually players are referred for a surgical opinion when they are not responding to non-operative management. Data from all such patients undergoing surgery are prospectively documented in a standardized database and all arthroscopic procedures are systematically recorded on DVD. The clinical examination and arthroscopies were all performed by one senior Consultant Orthopaedic surgeon. Besides, a thorough history on gender, age, type and severity of injury, location of symptoms, duration of symptoms, mode of injury, previous ankle injuries, participation and level of sport and previous treatment, clinical tests were performed. Outcome of laxity tests, effusion, joint line tenderness, anterior impingement test and ROM were compared with non-injured side. Indications for ankle arthroscopy are persistent pain and/or effusion disabling from full performance in sports despite physiotherapy and rest. Arthroscopy is undertaken as day case procedure using a 2.9 mm ankle arthroscope with standard anterior lateral and anterior medial portals and appropriate instruments (Arthrex Inc, USA). All compartments (medial malleoli, talar neck, talar dome, lateral, posterior and medial ligaments and lateral malleoli) are systematically examined and probed and all pathology is documented on digital prints and live video. The purpose of this study was primarily to identify and study symptomatic cartilage injuries. However, some cases which included associated minor ligament damages (TFA or FC ligament injuries) which had been healed and had not caused functional instability were also included. Cartilage injuries were documented from location, size and severity using Outerbridge scale (1–4) by the surgeon. Patients with associated multi-ligament injuries, complete or partial syndesmosis tears, previous severe ligament damage, functional instability and patients with current fractures were excluded from the study. Multi-ligament injury was defined as damages to more than two ligaments. 61 patients out of 197 operated during the study period 2002–2005 fulfilled our inclusion criteria.

MRI is not done routinely by our centre for these injuries, but had been performed pre-operatively by the Team Medics on 26/61 players before attending our centre. These had been performed at various locations around the UK and had been interpreted by different radiologists in different hospitals. The MR scanners used therefore differed and sequences used for each scan varied. In many cases, they had been interpreted as normal which triggered us to investigate this issue further. To correct for interpreter error, MRIs were thus traced (20/26 could be found) and re-interpreted by an experienced blinded musculoskeletal radiologist who was informed of the clinical symptoms of each patient, but blinded to the fact that the patients had been shown to have chondral lesions on arthroscopy. To further minimize bias, two scans were included, which involved ligament injury but no cartilage damage. The machine and sequences used for each patient were recorded and tabulated. 'Conventional' imaging for these cases was T1 and T2 weighted spin-echo or fast spin echo and gradient-echo sequences used included fat suppression and STIR (short inversion time recovery). The cartilage specific sequences involved fat suppressed 3D SPGR methods. For each sequence and plane, the TR, TE, FOV, resolution, slice thickness, scan time, number of acquisitions and result was noted and then tabulated. At the time of interpretation, the musculoskeletal radiologist and another unbiased consultant orthopaedic surgeon also explored the presence, size, location and depth of subchondral oedema. The sagittal slice with the deepest area of oedema was chosen and kept constant for each patient. Using a light box and plain white paper, the depth was measured. The paper was placed on the joint line and a mark was made where the oedema disappeared. The mark was made parallel to the joint line and the reading was taken perpendicular to the two lines. The perpendicular line was measured using a ruler and then measured on the scale for the specific scan. The oedema was measured on each sequence in order to firstly investigate whether certain sequences were better at delineating the oedema than others and secondly, to work out whether oedema looked deeper on some sequences rather than others. Results were only taken from the sequence with the deepest oedema for consistency. The interpreters made 3 readings in order to gain more accurate results. The mean depth was then worked out for each sequence. The depth was the converted to a grade. The system was: < 0.5 cm = grade 1, 0.5 to < 1.0 cm = grade 2, 1.0 cm to < 1.5 cm = grade 3, 1.5 cm and above = grade 4

An unbiased observer, not involved in the treatment of these patients, tried to contact all operated patients for a clinical follow up using telephone interview and a questionnaire. The questionnaire was constructed to evaluate the subjective outcome of surgery and return to sport. The average follow-up time was 24 months (range 10– 48 months). One patient was contacted 10 months after surgery, 20/61 patients between 12 and 24 months, and 30 patients after more than 24 months. Ten players could not be reached.

At the end of the study period, the data base, all patients' files and other documentation were systematically audited, evaluated and analyzed using SPSS statistical programme by an unbiased observer not involved in the treatment of the patients. Informed consent was given. The data was anonymized through coding. The study was approved by Sheffield University Ethical Committee. Each variable was investigated and simple statistical tests were employed to look for correlations (Pearson's correlation coefficient). One way ANOVA's were used to compare means, and odds ratios were implemented to look at the probability of certain results occurring.

## Results

The majority of subjects were male (n = 45, 74%). The mean age was 30 years (27–33 years). Injuries were found to affect both ankles equally. The average duration of symptoms was 7 months (5.7–7.9 months) for 58/61 cases, and 3 other cases were operated within 3 weeks from injury due to severe ankle pain and haemarthrosis after an ankle injury during a game, where X ray excluded fracture. Video recordings from the latter three cases showed a direct impact to the ankle from the opponent's studs or shoes in tackle situations, but the injuries were referred as "sprains" by the Team medics. All players had undergone "physiotherapy" as stated by referrals, but the questionnaire revealed that 41/61 players (68%) had received active physiotherapy including proprioceptive and strength training and local symptomatic treatment, whilst the rest had been advised by the physiotherapist either to rest for varying time periods or in some cases, to try to continue to play despite symptoms. 26/61 players (43%) were full-time professionals whilst 35 (57%) were semi professionals on different levels, 49% were footballers and 14% played rugby. 17/26 professional athletes (65%) played at international top level. The remaining athletes participated in either athletics, gymnastics, golf, cricket, tennis, netball, orienteering, lacrosse or hockey. 51/61 players (84%) injured their ankle during a competition/game. Over one-third of the patients had sprained their ankle and 7% had fractured their ankle at a previous occasion but played symptom-free until the index injury. 57/61 athletes (93%) complained of ankle pain as their predominant symptom at pre-surgery consultation. The majority of these players complained of a diffused exertion ankle pain, but some could point to an exact location.

40/61 (65%) had undergone an x-ray before arthroscopy on clinical suspicion of fracture, all deemed normal, and 26/61 (43%) had undergone an MRI. Of the 26 scans taken, 15/26 (59%) were reported as normal by the first radiologist and chondral injuries were identified in 5/26 cases. 20/26 scans were retraced and reinterpreted by an unbiased blinded musculoskeletal radiologist who identified chondral injuries in 10/20 cases. 3/20 scans presented minor osteochondral injuries as also revealed by arthroscopy. The detection rate of the chondral injuries was 3/17 (19%) for the first and 7/17 (41%) for the second radiologist. The results are shown in Table 4.

There was no significant correlation between the presence of oedema and the time period between injury and imaging. The grade of cartilage injury correlated significantly with the depth of oedema. All measurements were within one grade of each other apart from two grade 1 injuries where the oedema seemed to be deeper than we would have expected. (Table 5)

During arthroscopy, 8/61 players (13%) were found to have an isolated cartilage flap tear lesion with no other pathology. Of the remaining patients with chondral lesions, 21/61 (34%) presented with loose bodies, 6/61 (10%) occurred with osteophytes, 21/61 (34%) had associated minor ligament damage, which could be vizualised (FC ligament ruptures) and fibrosis whilst 5/61 (8%) occurred with a healed old un-displaced fracture. There were 75 cartilage lesions in the 61 patients presented in Table 2 and 3. 50% of the Talus dome lesions were grade IV lesions, 13.3% were grade III, 16.7% were grade II and 20% were grade I.

**Table 1:** list of subjective symptoms and clinical findings presented by the players, out of which pain, joint line tenderness and effusion were common denominators.

**Subjective Symptoms**	**Number of players**	**Percentage (%)**
Pain	58/61	95
Swelling	15/61	25
Stiffness	2/61	3
Instability	12/61	20
Clicking or locking	8/61	13
**Clinical findings**		
Joint line tenderness	56/61	92
Effusion	46/61	75
Decreased ROM	24/61	39
Clicking on passive movement	3/61	5
+ Anterior drawer/Talar tilt test	29/61	48
+ Anterior impingement test	19/61	31

Instability was claimed by 12/61 players, but only as secondary minor symptoms to their main complaint which was pain, thus passing our inclusion criteria. It is remarkable that 48% of the players had positive anterior drawer and talar tilt tests, whilst only 20% had any symptoms of instability at all and none of them claimed this was a major problem for them. Furthermore, even though only 25% complained of swelling, effusion was observed in 75% of the ankles.

**Table 2:** shows the number (n = 75) and locations of chondral lesions found at ankle arthroscopy in 61 patients.

Medial Malleolus	17
Tibial Plafond	6
Lateral Malleolus	0
Talar Dome	52

**Table 3:** shows the number (n = 52) and location of the Talar Dome cartilage lesions detected by arthroscopy in 61 patients.

Anterior Medial	18
Posterior Medial	9
Anterior Lateral	14
Posterior Lateral	3
Mid Talus Dome	8

Some lesions are slightly overlapping in areas but the areas that are mainly affected are presented below.

**Table 4:** demonstrates presence (Yes) of cartilage lesions (1a and 2a) and presence of subchondral oedema (Yes) (1b and 2b) detected on MRI by two experienced readers.

	**Reader 1a**	**Reader 2a**	**Reader 1b**	**Reader 2b**
Case 1	No	No	No	No
Case 2	No	Yes	Yes	Yes
Case 3	No	No	No	No
Case 4	No	No	No	No
Case 5*	Yes	Yes	Yes	Yes
Case 6*	Yes	Yes	Yes	Yes
Case 7	No	No	No	No
Case 8	No	No	No	No
Case 9	No	No	Yes	Yes
Case 10	No	No	No	No
Case 11	Yes	No	No	No
Case 12	Yes	Yes	Yes	Yes
Case 13	Yes	Yes	Yes	Yes
Case 14*	No	Yes	Yes	Yes
Case 15	No	No	Yes	Yes
Case 16	No	No	Yes	Yes
Case 17	No	No	No	No
Case 18	No	No	No	No
Case 19	No	No	Yes	Yes
Case 20	No	No	Yes	Yes

Case 5, 6 and 14 were the osteochondral injuries with minor bony component.

**Table 5:** shows a summary of the average depth of oedema, grade of lesion on MRI and at arthroscopy and the type of MR sequences used (n = 10)

**Outerbridge grade on arthroscopy**	**Average depth of oedema (cm)**	**Grade (on MRI)**	**Sagittal sequence used for measurement**
4	1.00	3	T2 Turbo spin-echo
4	2.58	4	Fat saturated Fast spin-echo
4	2.17	4	Fat saturated Fast spin-echo
4	1.50	3	Short time inversion recovery (STIR)
4	1.48	3	T2 SE
3	0.95	2	Fast spin-echo
2	1.47	3	Fat saturated Fast spin-echo
1	1.25	3	Spin-echo
1	1.00	2	T2 Turbo spin-scho
1	1.40	3	Fat saturated Fast spin-echo

During arthroscopy, chondral injuries were debrided, trimmed, vaporized or excised where deemed appropriate. Loose bodies were excised. Grade IV injuries were micro fractured. In no case, ligament reconstruction was undertaken. Immediate weight bearing was allowed. No immobilization was used.

At follow up, we managed to interview 51/61 players (84%) who also returned the questionnaires. Some of the missing players who were on short-term contracts in the UK had moved abroad and could not be traced. The average follow-up from arthroscopy was 24 months (range 10–48). One patient had a follow up 10 months after surgery, 20/61 patients were between 12 and 24 months, and 30 patients had more than 24 months follow-ups. The majority of athletes (37/51, 73%) were playing at the same high level of sport as prior to their injury. 24% (12/51) were playing at a lower level, and 2 players had ended their careers. The return to sport after surgery was in average 16 weeks (3–32 weeks). 22/61 players (36%) were still having some problems with their ankle. Out of these players, 12/22 patients (54%) experienced exertion pain as the predominant symptom. The remaining 10 (46%) complained of a mix of occasional pain, swelling and stiffness, occasional instability and occasional clicking and locking. 15/17 of the full-time professional international players were, with two exceptions (one rugby player, one footballer), still playing at their pre-injury level. The two exceptions are now playing at a lower level. Two previously semi-professional players ended their careers altogether due to this ankle injury.

## Discussion

We suggest that arthroscopy should be considered early in athletes with clinical presentation of exertion ankle pain, effusion and palpation tenderness along the joint line after a previous ankle sprain. The definition of "early" could of course be argued, but 7 months is certainly a long delay for a professional or semi professional player. In fact, most full-time professional players in this study (14/17) had even longer than average 7 months duration from injury to arthroscopy (10–36 months). A number of these cases were high profiled professional players who said that they had struggled for long time but "put up with the pain" with aid of symptomatic treatment. In most of these cases, the team medics often referred to a "normal MRI" as an important delaying factor, but also lack of definite diagnosis assumed to be a "low grade sprain", pressure to play from managers and financial implications of a long absence from playing were discussed. Only 3 international footballers were operated early (within 3 weeks from injury) due to severe pain and hemarthrosis and video recorded injuries demonstrating a substantial direct impact to the ankle from opponent's shoes or studs in tackle situations. They all had undergone MRI showing effusion with no chondral injuries, but were found to have major full thickness cartilage tears on arthroscopy. They were all back to play within 8 weeks from surgery, which is shorter than the average of 16 weeks for the other more chronic injuries.

Arthroscopic treatment was successful in a majority (73%) of all the studied players, and 15/17 professional top players could resume full play after surgery within reasonable time. In two cases, these injuries obviously ended their career. The long duration of symptoms prior to appropriate diagnosis and treatment could well be suspected to have aggravated the outcome of treatment. Since all the patients in this study were athletes, performance from injury to treatment was also likely to have been affected, even though we lack detailed information. Secondly, the fact that 36% of the players still had some degree of discomfort despite treatment may suggest that some of these injuries may turn into chronic problems and, possibly in the long term, osteoarthritis. An ankle arthroscopy is a relatively straight-forward procedure, which can be done as an outpatient's procedure, [3,7,8] and its complications from arthroscopy are rare [3].

The majority of cartilage lesions in our study were located on the Talus dome, which corresponds to other studies [1,3,6]. This can, to some extent, be explained by the work of Athanasiou et al. [13] who pointed out differences in the mechanical properties of specific human cartilage regions. For example, we know that the Tibia cartilage is stiffer than that of the Talus. The anterior and posterior regions of the lateral and medial sites of the tibia were found to be 18–37% stiffer than the anatomically corresponding sites in the Talus. Our findings correspond with Hintermann et al [1] and Hirose et al [14] who found that 62% of cartilage lesions in his study were on the medial aspect of the Talus. Only 17% were found on the lateral part of Talus. Some patients in our study had minor lateral ligament damage as well but stated that instability was a minor problem compared with the pain they experienced. Isolated chondral lesions may not be as common as lesions associated with ligament or bony damage. However, it is possible that our cases may include sprains as well as sprain-direct impact injuries, as demonstrated from some video analyses from the injury made available for us. There are unfortunately no systematic data available on this, but it certainly warrants further investigations. It is logical to assume that direct stud or shoe impact injuries to the ankle in a tackle situation can cause chondral damages as well as ligament tears from the pure impact and we suggest this mechanism as an important explanation for chondral injuries of the ankle as well as biomechanical factors included in sprains as discussed by others [16]. Van Dijk et al [15] found an association between the rupture of the lateral ligaments and medial joint pain. They found 19 cartilage lesions located on the medial malleoli or medial talus in 30 patients with lateral ligament ruptures. These results can be explained by Noguchi et al [16]. They showed that an increase of stress distribution on the medial side of the ankle joint occurred when the lateral ligaments were released. Harrington [17], on the other hand, suggested unbalanced loading of the medial joint space as be the primary cause for the development of degenerative arthritis. As the Tibia cartilage on the medial and posterior sides is 18–37% stiffer than its corresponding Talus sites, even minimal impact can cause damage to the cartilage of the medial Talus dome [13].

Within the limitations of our sample size, the use of conventional MRI to delineate articular cartilage injuries must be argued, in particular since 50% of the talar dome cartilage lesions were full thickness tears. Whilst both the initial and second radiologists agreed completely on the presence of subchondral oedema, the presence of chondral injuries was more controversial (19% versus 41%). Nevertheless, both of these figures are inadequate for clinical practice. The only sequences that accurately depicted a chondral lesion were the sagittal T2 Turbo spin echo (TSE), T1 spin echo (SE), fat saturated fast spin echo (FSE), T1 FSE and coronal fat sat FSE. We noted that all patients had an axial scan. The only sequence that accurately and clearly delineated the cartilage of the talus was the T2 TSE. The fast spin echo was no better than the conventional spin echo. MRI of articular cartilage is challenging because most articular surfaces are curved and thin and images are also prone to artefacts. The closely applied joint surfaces with thin cartilage and complex osseous anatomy make conditions challenging for the radiologist [4,6]. We speculate that the inaccuracies of the scans are due to the fact that the majority of sequences can not define the cartilage from the subchondral bone. The cartilage is either the same intensity as the bone or fluid. Secondly, the slices are too thick. Images were usually 3 mm thick, taken at 1 mm intervals. One scan was actually taken with 4 mm slices. As the cartilage in the ankle is so thin, these slices are far too thick to accurately delineate a lesion. A higher spatial resolution is therefore needed. In the ankle where there is minimal fat, suppression is not needed and results in fluid and cartilage producing the same signal intensity. It should be stressed that we do not know the initial detailed request to the MRI department by the referring Team doctor, which could of course influence the choice of sequences. Our findings correlate with the majority of authors who have studied the usefulness of MRI in the detection of similar lesions in the knee. Friemart stated that the sensitivity of detecting cartilage lesions in the knee by MRI can range from 15% to 96% [18]. Rubin et al demonstrated that the presence of subchondral oedema in the knee may be indicative of a defect in the overlying articular surface [12]. Even small defects of the articular surface were frequently associated with relatively large areas of subchondral marrow oedema. This seems to apply also for ankle injuries. The depth of oedema should also be taken into account as generally the deeper the oedema the more severe the cartilage lesion. More research is needed in this field. A technique that reliably detects cartilage lesions will greatly aid in the diagnosis and outcome of these injuries [19-22].

In conclusion, chondral injuries to the talo crural joint shows rather characteristic clinical symptoms and signs including subjective exertion pain, effusion and joint line tenderness on palpation and may occur with or without clinical signs of ligament laxity. In such cases, even with a "normal MRI" or an MRI showing subchondral oedema, an early arthroscopy should be considered. We suggest that the mechanism of some of these injuries in contact sports may involve direct impact to the ankle with or without sprain, which warrants further studies.
